# Dataset on wood density of trees in ecotone forests in Northern Brazilian Amazonia

**DOI:** 10.1016/j.dib.2020.105378

**Published:** 2020-03-07

**Authors:** Hugo Leonardo Sousa Farias, Williamar Rodrigues Silva, Ricardo de Oliveira Perdiz, Arthur Camurça Citó, Lidiany Camila da Silva Carvalho, Reinaldo Imbrozio Barbosa

**Affiliations:** a UFRR/PRONAT, Boa Vista, Roraima, Brazil; b INPA/PPGBOT, Boa Vista, Roraima, Brazil; c INPA/NAPRR, Boa Vista, Roraima, Brazil; d University of Exeter, Exeter, UK

**Keywords:** Specific gravity, Forest ecology, Tropical forest, Tree, Wood technology

## Abstract

Wood density is expressed by the ratio between dry weight and fresh volume of a sample piece. The value of this measure is an important variable for assessing wood functional properties, successional stages and biomass/carbon stock estimates in different terrestrial ecosystems. Wood density data were collected for tree species from ecotone forests of the northern Brazilian Amazonia. We sampled 680 individuals with stem diameter ≥10 cm. For each sampled individual measurements were taken for three stem variables: bark thickness (mm), bark density (g cm^−3^) and wood density (g cm^−3^). This dataset is intended to improve biomass and carbon estimates of forests in the northern ecotone region of Brazilian Amazonia, an area poorly known in terms of ecosystem dynamics.

**Table:** **Specification Table**

Subject	Agricultural and Biological Sciences

Specific subject area	Forestry and Plant Science

Type of data	Table and figure

How data were acquired	To obtain samples, an increment borer (Haglof Borer Auger), 150 mm in length and 5.15 mm in diameter, was used. Laboratory analyzes used precision scales (0.001 g) and an electric oven.

Data format	Raw and analysed

Parameters for data collection	Data collection only considered trees of stem diameter ≥10 cm, dispersed within 129 sampling plots, as a part of the research grid of the Biodiversity Research Program (PPBio) installed on the eastern side of Maracá Island, northern Brazilian Amazonia

Description of data collection	Laboratory analysis of collected samples used as a reference for the calculation of the wood density (g cm^−3^) the ratio of sample dry mass (g) divided by its saturated volume (cm^3^) for three stem variables: bark thickness, bark density, wood density (sapwood + heartwood). Saturated volume of each sample was estimated from a wood sample immersed in distilled water in a graduated cylinder (precision scale = 0.001 g). Weight was measured when the inserted sample was considered equal to the displaced volume, taking water density to be 1 g cm^−3^. Samples were then oven dried at 103 ± 2°C until a constant weight was achieved (∼72 hours).

Data accessibility	Repository name: Mendeley Data Data identification number: 5 Direct URL to data: http://dx.doi.org/10.17632/n4kzj3d2g7.5
	

## Value of the Data

• A wood density database is essential to improve biomass and carbon stock estimates at local, regional and global scales.
• The generated data are key for understanding of climate change effects on ecotonal forest dynamics in northern Brazilian Amazonia.
• These data are an important reference source for research on tree species functional traits linked to diversity and spatial distribution.

## 1 Data

**Fig. 1:**
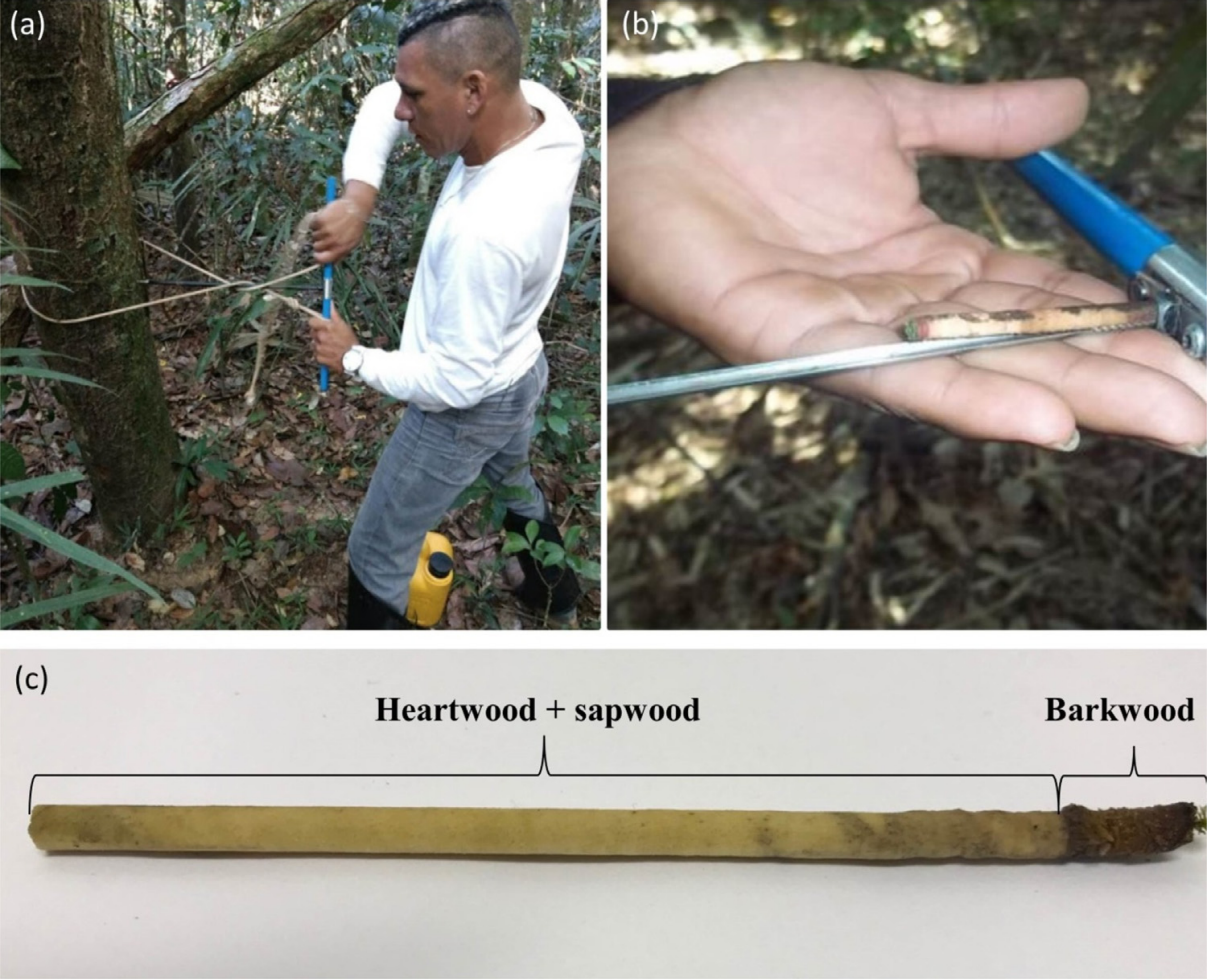
Fieldwork: (a) collection of stem samples using an increment borer; (b) detail of the sample taken from the stem and (c) subdivisions of the samples that were considered in this research for calculation of wood density.

This research reports on a wood density data set for northern Brazilian Amazonia ecotonal forests. Table 1 shows the density of stem wood (sapwood + heartwood), bark density and bark thickness from 110 tree species and morphospecies (mean ± SD) present in ecotone forests of eastern Maracá Island. Fig. 1 shows the fieldwork to collect the stem samples and the subdivisions of the samples considered in this research for calculation of wood density.

**Table 1:** Tree species and morphospecies wood density from ecotone forests of northern Brazilian Amazonia (mean ± SD). Samples = number of individuals sampled, Bark T = bark thickness in millimeters, Bark D = bark density, Core WD = sapwood + heartwood density, Weighted average WD = weighted average between Bark D and Core WD.

Family	Species	Samples	Bark T (mm)	Bark D (g cm^−3^)	Core WD (g cm^−3^)	Weighted average WD (g cm^−3^)
Achariaceae	*Lindackeria paludosa*	3	3.74 ± 1.26	0.606 ± 0.139	0.694 ± 0.101	0.691 ± 0.094
Anacardiaceae	*Astronium lecointei*	3	6.00 ± 2.65	0.691 ± 0.076	0.778 ± 0.178	0.774 ± 0.170
	*Spondias mombin*	1	14.1	0.250	0.774	0.735
Annonaceae	*Duguetia lepidota*	14	8.14 ± 2.21	0.535 ± 0.102	0.796 ± 0.043	0.780 ± 0.042
	*Duguetia lucida*	3	10.33 ± 4.04	0.407 ± 0.129	0.732 ± 0.023	0.710 ± 0.020
	*Guatteria citriodora*	1	4.1	0.128	0.604	0.594
	*Guatteria schomburgkiana*	8	8.27 ± 4.11	0.488 ± 0.177	0.646 ± 0.116	0.638 ± 0.113
	*Xylopia amazonica*	2	9.00 ± 0.00	0.533 ± 0.131	0.669 ± 0.093	0.662 ± 0.081
Apocynaceae	*Aspidosperma nitidum*	1	2.7	0.418	0.828	0.820
	*Aspidosperma spruceanum*	3	8.94 ± 2.54	0.733 ± 0.099	0.750 ± 0.024	0.750 ± 0.024
	*Himatanthus articulatus*	35	9.33 ± 4.40	0.459 ± 0.153	0.567 ± 0.039	0.562 ± 0.040
Araliaceae	*Schefflera morototoni*	2	3.50 ± 0.57	0.479 ± 0.086	0.323 ± 0.017	0.327 ± 0.019
Bignoniaceae	*Handroanthus obscurus*	2	5.00 ± 1.41	0.259 ± 0.058	0.862 ± 0.059	0.838 ± 0.064
	*Handroanthus uleanus*	4	4.45 ± 053	0.508 ± 0.104	0.811 ± 0.089	0.802 ± 0.086
Bixaceae	*Cochlospermum orinocense*	3	12.37 ± 4.75	0.520 ± 0.318	0.424 ± 0.147	0.425 ± 0.150
Boraginaceae	*Cordia tetrandra*	5	6.94 ± 2.91	0.441 ± 0.186	0.476 ± 0.200	0.476 ± 0.197
Burseraceae	*Protium neglectum*	2	3.50 ± 2.12	0.488 ± 0.387	0.554 ± 0.023	0.556 ± 0.014
	*Protium polybotryum*	2	4.94 ± 2.17	0.801 ± 0.212	0.571 ± 0.016	0.584 ± 0.001
	*Protium rhoifolium*	4	4.39 ± 3.19	0.701 ± 0.088	0.585 ± 0.045	0.589 ± 0.045
	*Protium stevensonii*	22	4.62 ± 2.11	0.705 ± 0.155	0.709 ± 0.072	0.709 ± 0.071
	*Protium unifoliolatum*	8	4.20 ± 1.68	0.614 ± 0.154	0.692 ± 0.049	0.688 ± 0.046
	*Trattinnickia glaziovii*	5	4.06 ± 1.47	0.624 ± 0.191	0.422 ± 0.024	0.427 ± 0.019
	*Trattinnickia rhoifolia*	3	5.81 ± 1.28	0.537 ± 0.017	0.521 ± 0.099	0.523 ± 0.094
Caryocaraceae	*Caryocar villosum*	1	6.8	0.707	0.569	0.575
Celastraceae	*Maytenus guyanensis*	5	3.08 ± 1.12	0.757 ± 0.128	0.722 ± 0.040	0.723 ± 0.040
Chrysobalanaceae	*Exellodendron barbatum*	8	3.71 ± 1.43	0.826 ± 0.116	0.841 ± 0.061	0.841 ± 0.061
	*Hirtela racemosa*	1	2.0	0.859	0.785	0.786
	*Leptobalanus apetalus*	5	3.30 ± 1.48	0.725 ± 0.123	0.747 ± 0.062	0.746 ± 0.063
Chrysobalanaceae	*Licania kunthiana*	3	3.96 ± 2.10	0.733 ± 0.055	0.803 ± 0.101	0.802 ± 0.097
	*Licania discolor*	17	5.09 ± 2.33	0.748 ± 0.176	0.825 ± 0.124	0.824 ± 0.121
	*Moquilea minutiflora*	3	6.50 ± 2.60	0.601 ± 0.067	0.624 ± 0.023	0.623 ± 0.019
Clusiaceae	*Garcinia macrophylla*	1	4.0	0.962	0.674	0.685
Elaeocarpaceae	*Sloanea guianensis*	2	4.00 ± 0.00	0.573 ± 0.348	0.870 ± 0.058	0.866 ± 0.061
Erythroxylaceae	*Erythroxylum mucronatum*	1	8.0	0.582	0.819	0.806
Euphorbiaceae	*Mabea speciosa*	2	2.82 ± 1.67	0.515 ± 0.546	0.567 ± 0.021	0.567 ± 0.030
Lamiaceae	*Vitex schomburgkiana*	3	5.47 ± 2.78	0.667 ± 0.074	0.606 ± 0.064	0.607 ± 0.063
Lauraceae	*Aniba* sp.	1	7.7	0.507	0.622	0.616
	*Endlicheria dictifarinosa*	1	10.0	0.565	0.478	0.483
	*Licaria chrysophylla*	1	2.0	0.988	0.677	0.682
	*Mezilaurus crassiramea*	3	4.66 ± 3.06	0.541 ± 0.213	0.697 ± 0.021	0.694 ± 0.024
	*Ocotea sandwithii*	7	4.31 ± 1.33	0.649 ± 0.245	0.664 ± 0.045	0.664 ± 0.041
Lecythidaceae	*Couratari multiflora*	1	4.1	0.203	0.468	0.459
	*Eschweilera pedicellata*	4	5.90 ± 3.48	0.767 ± 0.115	0.759 ± 0.035	0.758 ± 0.036
	*Eschweilera* sp.^1^	9	5.74 ± 3.24	0.603 ± 0.208	0.698 ± 0.141	0.695 ± 0.139
	*Gustavia augusta*	2	6.25 ± 0.95	0.340 ± 0.158	0.698 ± 0.037	0.682 ± 0.042
	*Lecythis corrugata* subsp*. rosea*	66	6.39 ± 3.14	0.628 ± 0.159	0.733 ± 0.074	0.730 ± 0.073
Leguminosae	*Albizia glabripetala*	1	4.5	0.398	0.622	0.617
	*Albizia pedicellaris*	1	9.0	0.598	0.405	0.411
	*Albizia* sp.	1	8.0	0.258	0.518	0.503
	*Andira surinamensis*	2	4.00 ± 0.00	0.413 ± 0.275	0.688 ± 0.037	0.682 ± 0.041
	*Caesalpinia* sp.	2	5.01 ± 4.26	0.561 ± 0.095	0.665 ± 0.057	0.660 ±0.050
	*Centrolobium paraense*	2	4.45 ± 0.64	0.843 ± 0.019	0.755 ± 0.004	0.756 ± 0.004
	*Dialium guianense*	1	0.5	0.746	0.784	0.784
	*Enterolobium schomburgkii*	2	4.00 ± 0.00	0.688 ± 0.080	0.573 ± 0.079	0.576 ± 0.074
	*Hymenaea* sp.	1	3.0	0.924	0.884	0.885
	*Inga splendens*	4	6.79 ± 1.20	0.570 ± 0.054	0.639 ± 0.070	0.636 ± 0.068
	*Inga cinnamomea*	1	4.5	0.656	0.525	0.530
	*Inga* sp.^2^	2	3.81 ± 3.26	0.722 ± 0.172	0.727 ± 0.006	0.727 ± 0.010
	*Ormosia coarctata*	2	5.16 ± 1.19	0.612 ± 0.232	0.822 ± 0.236	0.816 ± 0.239
	*Peltogyne gracilipes*	36	3.74 ± 2.06	0.841 ± 0.165	0.903 ± 0.091	0.901 ± 0.088
	*Peltogyne paniculata*	4	2.02 ± 1.18	0.922 ± 0.202	0.921 ± 0.037	0.922 ± 0.037
	*Swartzia grandifolia*	2	10.00 ± 9.90	0.513 ± 0.204	0.602 ± 0.245	0.599 ± 0.245
	*Swartzia latifolia*	1	5.0	0.451	0.694	0.684
	*Swartzia* sp.	1	4.2	0.699	0.778	0.775
Malpighiaceae	*Byrsonima schomburgkiana*	5	8.46 ± 3.75	0.616 ± 0.172	0.626 ± 0.150	0.626 ± 0.146
Malvaceae	*Apeiba tibourbou*	6	10.83 ± 4.48	0.353 ± 0.070	0.345 ± 0.124	0.348 ± 0.115
	*Luehea speciosa*	7	7.84 ± 2.53	0.501 ± 0.130	0.639 ± 0.063	0.631 ± 0.066
	*Pochota fendleri*	2	11.50 ± 3.54	0.324 ± 0.055	0.367 ± 0.035	0.364 ± 0.028
Melastomataceae	*Miconia stenostachya*	1	6.3	0.833	0.817	0.818
Meliaceae	*Trichilia cipo*	9	5.22 ± 2.24	0.723 ± 0.151	0.725 ± 0.054	0.725 ± 0.053
Moraceae	*Brosimum guianense*	5	5.41 ± 2.62	0.697 ± 0.201	0.768 ± 0.096	0.765 ± 0.097
	*Clarisia racemosa*	3	7.94 ± 8.87	0.806 ± 0.191	0.675 ± 0.045	0.676 ± 0.043
	*Pseudolmedia laevigata*	17	4.06 ± 1.38	0.642 ± 0.160	0.673 ± 0.057	0.672 ± 0.057
Myristicaceae	*Virola calophylla.*	2	8.05 ± 1.48	0.582 ± 0.155	0.591 ± 0.009	0.591 ± 0.013
Myrtaceae	*Calyptranthes fasciculata*	1	5.0	0.660	0.797	0.791
	*Eugenia essequiboensis*	1	2.5	0.556	0.686	0.683
	*Eugenia omissa*	5	3.00 ± 1.58	0.640 ± 0.337	0.758 ± 0.064	0.757 ± 0.057
	*Psidium guineense*	1	2.0	0.861	0.829	0.830
Nyctaginaceae	*Neea oppositifolia*	1	13.7	0.507	0.543	0.541
Ochnaceae	*Quiina rhytidopus*	11	3.42 ± 1.55	0.663 ± 0.260	0.823 ± 0.063	0.819 ± 0.063
Olacaceae	*Chaunochiton kappleri*	2	10.00 ± 5.66	0.403 ± 0.073	0.616 ± 0.197	0.603 ± 0.186
Peraceae	*Pera bicolor*	1	2.8	0.787	0.803	0.803
Putranjivaceae	*Drypetes variabilis*	1	4.2	0.941	0.698	0.705
Rubiaceae	*Alseis latifolia*	33	3.55 ± 2.56	0.533 ± 0.219	0.645 ± 0.050	0.642 ± 0.048
	*Amaioua corymbosa*	4	4.41 ± 3.02	0.659 ± 0.293	0.726 ± 0.051	0.729 ± 0.053
	*Chomelia tenuiflora*	1	3.6	0.697	0.684	0.684
	*Duroia eriopila*	14	3.74 ± 2.32	0.577 ± 0.145	0.683 ± 0.074	0.681 ± 0.074
	*Guettarda macrantha*	3	4.70 ± 1.41	0.538 ± 0.191	0.541 ± 0.058	0.540 ± 0.059
	*Palicourea crocea*	1	6.5	0.557	0.624	0.621
	*Posoqueria latifolia*	1	1.0	0.736	0.552	0.554
	*Rudgea crassiloba*	5	2.58 ± 1.01	0.764 ± 0.278	0.647 ± 0.038	0.650 ± 0.035
	*Rudgea* sp.	2	3.68 ± 0.97	0.301 ± 0.130	0.575 ± 0.035	0.568 ± 0.029
Salicaceae	*Casearia spinencens*	1	1.0	0.645	0.588	0.589
	*Casearia sylvestris*	8	3.58 ± 0.55	0.482 ± 0.150	0.708 ± 0.066	0.701 ± 0.064
	*Xylosma benthamii*	1	5.0	0.317	0.697	0.685
Sapindaceae	*Cupania rubiginosa*	2	7.93 ± 1.51	0.653 ± 0.041	0.764 ± 0.011	0.758 ± 0.009
Sapotaceae	*Chrysophyllum sparsiflorum*	3	4.52 ± 0.50	0.855 ± 0.175	0.855 ± 0.034	0.856 ± 0.037
	*Ecclinusa guianensis*	70	6.46 ± 3.08	0.642 ± 0.166	0.661 ± 0.043	0.660 ± 0.043
	*Pouteria cuspidata*	3	4.24 ± 1.65	0.429 ± 0.063	0.717 ± 0.050	0.707 ± 0.053
	*Pouteria hispida*	16	3.52 ± 2.28	0.654 ± 0.183	0.818 ± 0.085	0.816 ± 0.083
	*Pouteria reticulata*	6	3.41 ± 1.39	0.649 ± 0.230	0.735 ± 0.042	0.733 ± 0.039
	*Pouteria* sp.	1	4.7	0.744	0.739	0.739
	*Pouteria surumuensis*	26	4.74 ± 1.47	0.540 ± 0.162	0.909 ± 0.081	0.898 ± 0.079
	*Pouteria venosa*	11	4.19 ± 1.95	0.596 ± 0.248	0.782 ± 0.084	0.777 ± 0.085
	*Pradosia surinamensis*	24	6.67 ± 3.08	0.477 ± 0.146	0.681 ± 0.046	0.673 ± 0.043
Simaroubaceae	*Simarouba amara*	10	7.93 ± 4.15	0.615 ± 0.199	0.422 ± 0.036	0.427 ± 0.034
Violaceae	*Leonia glycycarpa*	1	4.8	0.688	0.680	0.681
	*Rinorea pubiflora*	3	5.36 ± 3.00	0.503 ± 0.255	0.685 ± 0.052	0.680 ± 0.059

1 Mean of values for *Eschweilera* sp. 1 and *Eschweilera* sp. 2 morphospecies.

2 Mean of values for *Inga* sp.2, *Inga* sp.3 morphospecies.

## 2 Experimental design, materials and methods

### 2.1 Sampling area description

**Fig. 2:**
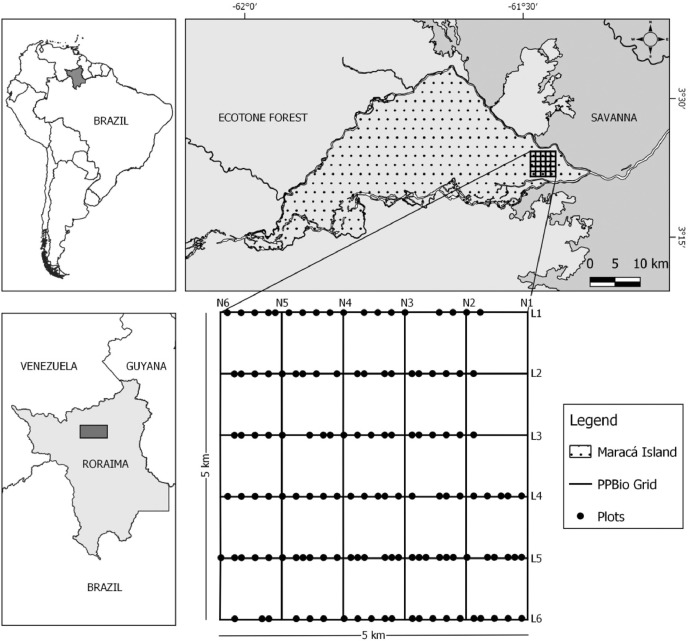
Localization of the PPBio research grid within the eastern part of Maracá Island.

Data were collected from the Biodiversity Research Program (PPBio) research grid, located on the east of Maracá Island (or Ilha de Maracá), which lies within the Maracá Ecological Station (3.360 N a 3.405 N / -61.442 W a -61.486 W), State of Roraima, northern Brazilian Amazonia, as showed in the Fig. 2. Maracá Island has an area of ∼101,000 ha, being 60 km long and some 15–25 km wide [1,2]. This region occupies the climatic transition between Köppen classification subtypes (Aw) and (Am), with annual average temperature of 26 °C and annual average precipitation of 2086 ± 428 mm. The wettest months (>300 mm month^−1^) are from May to August, and the driest from December to March (<100 mm month^−1^) [1], [2], [3], [4].

The vegetation of Maracá Island includes a variety of forest and non-forest types as the main feature of the savanna-forest transition zone of north central Roraima [2,^5^,6]. The different dominant forest types of the contact region are characterized by a mosaic of ombrophilous and seasonal forests (semideciduous and deciduous) whose composition and location are determined by distinct hydro-edaphic constraints, with the presence/absence of individuals of *Peltogyne gracilipes* Ducke (Leguminosae) operating as a robust environmental indicator [4,^7^,8]. Other technical details and environmental information on PPBio grid installed in Maracá Island can be accessed in the official PPBio website (https://ppbio.inpa.gov.br/sitios/maraca).

### 2.2 Sample processing and analysis

Field collection and construction of the current Dataset were derived from an existing forest inventory [8] carried out in the 25 km^2^ grid of PPBio (Biodiversity Research Program) installed on the eastern part of Maracá Island as described above. All samples to estimate the wood density of the different tree species occurring in the ecotone forests on eastern of Maracá Island were obtained from a systematic sampling of 129 plots (50 m x 10 m/6.45 ha in total) dispersed throughout the PPBio grid. These plots were intentionally established with small dimensions and with short between-plot distances to obtain high spatial resolution, and so better capture the microvariations in structural and species composition present across the island's altitudinal gradient; which defines the distinct hydro-edaphic conditions under which the different forest types of Maracá Island occur. The minimum distance between the plots was 150 m, based on the distance-markers located every 50 m along the PPBio grid trails; all sampling plots are georeferenced in UTM and with topographically defined altitudes. All data and metadata related to trail topography is available on the official PPBio website [9,10]. Plots in aquatic environments (swamps) and open areas enclaves (savannas) were discarded because they do not contain forest environments. The fieldwork was carried out in two stages: January / 2018 (269 samples) and January / 2019 (411 samples). Both fieldworks were carried out purposely at the peak of the regional dry period in order to avoid the variation of wood moisture due to climatic seasonality, and a possible bias in the biomass/carbon stock estimates.

## Appendix Supplementary materials

Image, application 1
